# Bonding efficacy of an acetone/based etch-and-rinse adhesive after dentin deproteinization

**DOI:** 10.4317/medoral.17717

**Published:** 2012-02-09

**Authors:** Fátima S. Aguilera, Raquel Osorio, Estrella Osorio, Pedro Moura, Manuel Toledano

**Affiliations:** 1 Department of Dental Materials, School of Dentistry, Campus de Cartuja 18071, University of Granada, Spain; 2 Department of Restorative Dentistry, Instituto Superior de Ciências da Saúde-Sul Egas Moniz, Campus Universitário Quinta da Granja, Monte da Caparica 2829-511, Portugal

## Abstract

Objectives: to evaluate the effect of sodium hypochlorite (NaOCl) treatment on dentin bonding by means of shear bond strength (SBS) measurements when using Prime&Bond NT (PB NT) adhesive. Ultrastructure of the interfaces was examined by scanning electron microscopy (SEM). Study design: Extracted human third molars were sectioned and ground to expose flat surfaces of superficial or deep dentin. Specimens were randomly assigned to two equal groups, and bonded as follows: (1) according to the manufacturers’ directions, after 35% H3PO4 etching, (2) 5% NaOCl treated for 2 minutes, after 35% H3PO4 etching. Each sample was embedded in a Watanabe shear test assembly for a single plane lap shear. After PB NT bonding, specimens were stored in water for 24 h at 37ºC and thermocycled (500x). Samples were tested in shear to failure using a universal testing machine at 0.75 mm/min. Data were analyzed with ANOVA and Newman-Keuls multiple comparison test procedures. Two samples of each group were randomly selected to investigate the morphologic aspect of the resin/dentin interface with SEM. Results: After etching and after aqueous sodium hypochlorite (NaOClaq) application, SBS values were similar on superficial than deep dentin (p>0.05). SEM findings shows for H3PO4 etching conditioned samples a detectable hybrid layer and long resin tags; for NaOCl treated specimens, it may be observed a non apparent hybrid layer, and the adhesive contact directly with the neck of the cylindrical resin tags. Conclusions: The use of 5% NaOCl for 2 min after dentin demineralization when PB NT was employed did not improve the bond strength to dentin, probably due to nanofiller content and/or oxidative changes on collagen-depleted dentin.

** Key words:**Sodium hypochlorite, shear bond strength, SEM, Prime&Bond NT, superficial dentin, deep dentin.

## Introduction

Etching of dentin removes the mineral phase and leaves the unsupported organic phase suspended in water, exposing the dentinal collagen matrix as a bonding substrate (allowing adhesive infiltration), and this is a safe and practical approach to improve bonding dentin (1). Nakabayashi et al. (2) demonstrated that the process of hybridization is believed to result from the infiltration of the primer into the open spatial network in the collagen matrix exposed by dentin demineralization and its in situ polymerization. Degradation may occur by (i) breakdown of the polymer phase (within the adhesive and the hybrid layers) or collagen fibrils in the hybrid layer, or (ii) exposure of collagen matrix of dentin by acid etching may also activate matrix metalloproteinase (MMPs) (1). To avoid this biodegradation different strategies have been proposed, such as the demineralized collagen removal (3,4) and the use of MMPs inhibitors (5).

Sodium hypochlorite (NaOCl) is a well-known nonspecific proteolytic agent capable of removing organic material (6). The proteolytic action of NaOCl is believed to involve extensive fragmentation of long peptide chains and formation of N-chloramines with terminal amine groups that further descompose to other byproducts, including inter- and intramolecular crosslinks via Schiff base formation (7,8). NaOCl-treated dentin is rich in exposed hydroxiapatite crystals (8) and could result in a more stable interface over time because it is essentially made of mineral (9). Depending on each testing methodology and/or specific composition of each dentin adhesive, the application of NaOCl upon etching may increase or decrease bond strengths (6).

Efficient diffusion of primers and resins, and saturation of spaces around dentin structures are essential to good dentin bonding, because this adhesion is carried out by polymerization of liquid monomers after their penetration on the dentin matrix (10). In adittion, the histological characterization of dentin shows that it is an inhomogeneous tissue as it is composed of intertubular and peritubular dentins, with different mineral content. The latter varies also in relation to dentin location (11,12). In consequence, the surface treatments may affect differently the superficial and deep dentin (i.e., deep dentin is a more hydrated substrate than superficial dentin after acid etching) (12). Therefore, changes in the dentin structure resulting from demineralization and NaOCl treatment due to differences in dentin depth could all influence spreading of various adhesive systems.

This study aimed to determine the effects of different conditioning procedures (H3PO4 and H3PO4+NaOCl) on shear bond strength and on ultrastructure of the resin-dentin interfaces with the use of scanning electron microscopy (SEM) of an acetone/based etch-and-rinse adhesive. The null hypothesis tested was that phosphoric acid and sodium hypochlorite pretreatments influence neither bond strength nor the resin-dentin interface ultramorphology of a one-bottle dentin adhesive system containing acetone to superficial and deep dentin.

## Material and Methods

-Specimen preparation

Forty eight caries-free extracted human third molars were stored in 0.5% chloramine T (Sigma-Aldrich, S.A., Madrid, Spain) at 4o C and were used for up to one month, as ISO standard 11405 recommends (13). Human specimens were obtained with the informed consent of donors, under a protocol that was reviewed and approved by the Institutional Ethics Committee. The teeth were cleaned of debris and mounted in phenolic rings with cold-cured acrylic resin, leaving the occlusal two-thirds of the crown exposed.

-Shear bond strength (SBS) test

The specimens were sectioned below the dentin-enamel junction ground flat and automatically polished up to 600-grit (Struers LaboPol-4, Struers, Copenhagen, Denmark) using silicon carbide papers under running water, during 60 s, to provide flat dentin surfaces. Half of the teeth were sectioned and ground, as stated above, to provide uniform surfaces of superficial dentin. Surfaces were observed in a stereomicroscope to ensure that no residual enamel was left. The rest of them were sectioned 1.1 ± 0.1 mm below the original level and ground flat, in order to expose deep dentin. Shear bond strength testing was performed according to ISO standard 11405 (13). The specimens were mounted on the Watanabe test device (9) with the roots and pulps of all the teeth remaining intact. Each specimen was embedded in die stone in one half of a Watanabe jib. All samples remained hydrated during these procedures, keeping excess water on the dentin surface preparations. After completion of the surfaces, the bonding system Prime & Bond NT (PB NT) and Tetric Ceram resin composite shade A3.5 were applied according to the manufacturer’s directions in 24 specimens (superficial dentin: n=12; deep dentin: n=12), following the procedure described by Toledano et al. (14). In others 24 specimens (superficial dentin: n=12; deep dentin: n=12), after applying and rinsing the acid conditioner, the dentin surface was treated with 5% aqueous sodium hypochlorite solution (NaOClaq) for 2 min with constant agitation and rinsed for 2 min with distilled water. The adhesive was then applied as described. After mounting, the assemblies were placed in water at 37ºC for 24 h and the thermocycled 500 times between water baths held at 5ºC and 55ºC with a dwell time in each bath of 30 s.

Shear testing was then conducted using an Instron Universal Testing Machine, model 4411 operating at a crosshead speed of 1 mm/minute. The bond strength values were calculated in Megapascals (MPa). Two-way ANOVA and Student t test were applied for comparisons at a corrected significance level of p<0.01.

The fractured specimens were examined with a stereomicroscope (Olympus SZ-CTV, Olympus, Tokyo, Japan) at 40x magnifications to determine the mode of failure. Failure modes were classified as adhesive (A) or mixed (M).

-Scanning Electron Microscopy (SEM) examination of the resin-dentin interface

Two specimens from each group were examined with a SEM (Zeiss DSM-950, Karl-Zeiss, Germany) to study the characteristics of the resin/dentin interface after different surface treatments. The specimens were cross-sectioned by means of the water-cooled diamond saw to produce 2-mm thick slabs. The prepared specimens were mechanically polished with wet, 600-, 1200- and 4000-grit silicon carbide papers. Final polishing was achieved using a diamond polishing-paper Struers LaboPol-4 (Struers; Copenhagen, Denmark) for 10 s. The polished specimens were ultrasonicated for 5 minutes in deionised water (model 512, P-Selecta, Barcelona, Spain) immersed in 95% ethanol, and gently air-dried. Each specimen was sputter-coated with gold-palladium (Unit E500, Polaron Equipment Ltd., Watford, England) and examined with a SEM at 20 kV to evaluate microscopic fracture patterns and the morphology of the resin/dentin interfaces.

Results

-Shear bond strength

 Table 1 displays mean and standard deviations of the different treatments and dentin depth. ANOVA showed that dentin depth and dentin treatment did not influence SBS if they were independently evaluated, and interactions were neither significant. After etching, SBS values were similar on superficial than deep dentin (p>0.05). After NaOClaq application, no statistically significant difference in SBS could be observed (p>0.05) for each deep dentin.

**Table 1:**
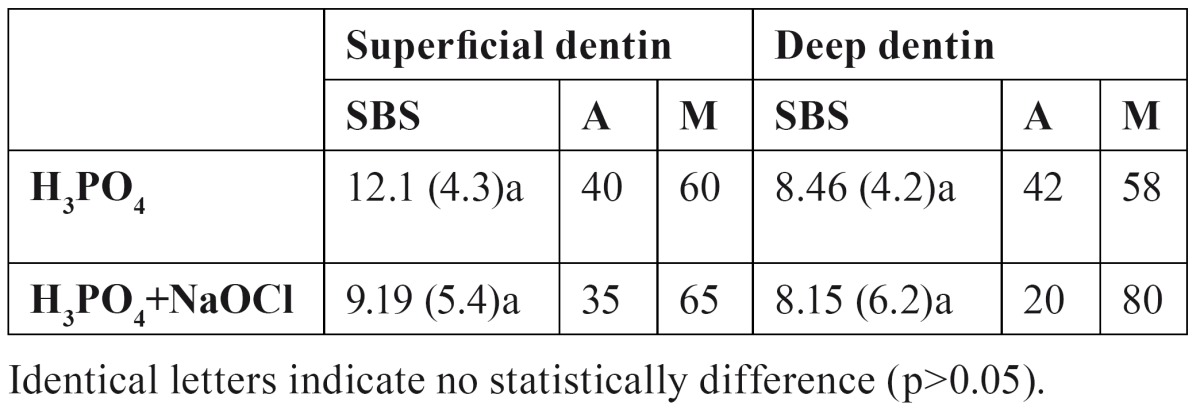
Mean and standard deviation (SD) shear bond strength (SBS) values (in MPa) obtained on superficial and deep dentin, after acid etching and acid/NaOCl application, and distribution of failure modes (%). (A: adhesive; M: mixed).

The percentage failure modes of the debonded specimens according to the surface conditioning are summarized in  Table 1. Mixed failures that involved partial dentin failures along the surface and partial failures within the adhesive/resin were normally associated with high bond strength values. The percentage of mixed failures increased with NaOClaq treatment in deep dentin.

-Scanning Electron Microscopy

**Figure 1:**
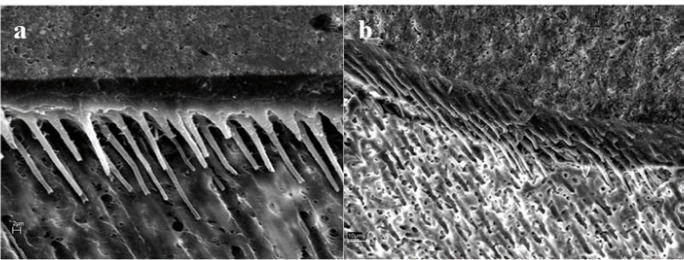
SEM image of dentin-resin interface when Prime & Bond NT (PB NT) was applied after H3PO4 application. a) For superficial dentin, a thin and uniform hybrid layer can be observed. Funnel-shaped and long resin tags with some lateral branches are evident (bar=2 µm). b) For deep dentin, wide and long resin tags were exhibited. These resin tags were also funnel-shaped, so the removal of the peritubular dentin by the acid treatment was evident. Numerous small lateral extensions of micro-tags branching off at right angles from the main resin tag were frequently observed (bar=10 µm).

**Figure 2:**
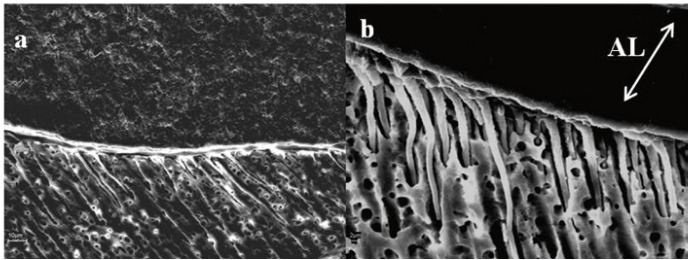
SEM image of dentin-resin interface when PB NT was applied after H3PO4 and NaOCl application. In both micrographs the hybrid layer was not apparent. a) For superficial dentin, few cylindrical resin tags were longer than that of Fig. 1a. Note the presence of resin tags whose necks contact directly with the adhesive or residual hybrid layer (bar=10 µm). b) For deep dentin, cylindrical and long resin tags were evident. These tags come virtually from the adhesive. (AL= Adhesive Layer) (bar=2 µm).

The SEM findings are summarized with micrographs in figures 1 and 2. When ortophosphoric acid etching was employed, a detectable hybrid layer was showed and many wide and long resin tags were exhibited, regardless of dentin depth (Fig. 1). These resin tags were funnel shaped. For etched and NaOCl treated specimens, a non apparent hybrid layer may be observed, and the adhesive may contact directly with the neck of the cylindrical resin tags (Fig. 2). Moreover, when the deep dentin was NaOCl treated the SEM image exhibited changes on underlying dentin (Fig. 2): the main tubule entrances on the peritubular and intertubular regions are widening.

## Discussion

The bonding interface consisted of multiple structures such as bonding resin, hybrid layer and dentin substrate (15). As for the bond to enamel and dentin, micromechanical adhesion is assumed to be the prime bonding mechanism. Moreover, diffusion and capillarity are the primary mechanisms to obtain micro-mechanical retention (10).

It was discussed whether bond strength tests are in gene-ral able to predict the clinical behavior of adhesively bonded composite resin restorations since they do not represent the complex clinical failure mechanism. In tensile tests, the fracture starts at the weakest part of the bond. A disadvantage of these tests is the high technique sensitivity (16). In a shear test the fracture does not start at the weakest part of the bond, but always at the insertion point of the load (17). The preference for conventional shear is justified because they are easy to perform, requiring minimal equipment and specimen preparation (16). SBS tests are the comparatively most simple test procedures and provide a reproducible starting point of loading (17). The influence of bonding area, on the other hand, remains undefined, though at least a trend for increasing bond strength values with the use of smaller bonding areas does exist (16). Therefore, we have employed the Watanabe SBS test in this study that was included as test methods for the adhesion of restorative materials to tooth structure into the ISO/TS 11405 (13).

Depth of dentin did not affect the bond strength of this etch-and-rinse adhesive system, according to other researchers (18), regardless surface pretreatment ( Table 1). The relative number of exposed tubules, the area of peritubular dentin, and the area occupied by intertubular dentin vary dramatically depending on the depth of dentins being observed (19). Gwinnett (11) held that the total bond strength of resin to dentin is the sum of individual bond strengths promoted by resin tag formation, hybrid layer, and surface adhesion. In deep dentin the width of the tubular apertures became greater. However there is less intertubular dentin and hence less surface adhesion than in superficial dentin (9). Moreover, the smaller the dentin thickness towards the pulp the larger is the percentage area of dentinal tubuli and the greater tubule area increases hydration (11). In addition, failure in removing all residual water entrapped in the deepest regions of demineralized surfaces, and demineralized and NaOClaq treated dentin induces the formation of poorly polymerized polymer chains (4). All these factors could equilibrate the SBS data of superficial and deep dentin.

After NaOClaq application, no statistically significant difference in SBS could be observed for each deep dentin. The removal of collagen did not play any role in the SBS obtained in dentin with PB NT. Other authors (9) did not obtain differences on bond strength between etched and etched/NaOCl treated dentin when some etch-and-rinse single-bottle adhesives were used. Thus, no relation between surface roughness and bond strength was made, as pointed out by other researchers (4). Nevertheless, the percentage of mixed failures increased with NaOClaq treatment in deep dentin ( Table 1). Several researchers (4,8) have studied the role of NaOCl in dentin permeability and in dentin adhesion. Depending on each testing methodology and/or specific composition of each dentin adhesive, the application of NaOCl upon etching may increase or decrease bond strengths (6,8,9). Based on bond strength data, it has been concluded that the higher the NaOCl concentration, the greater the bond strength until a plateau is reached at a concentration of 10% for an application time of 60 seconds (6,7).

Apparently, resin bonding to demineralized and NaOClaq treated dentin becomes similar to acid-etched enamel, with the tissue being less mineralized, more irregular, rougher and with unevenly distributed porosity (Fig. 2) (7,20). Mountouris et al. (7) demonstrated with Fourier Transform Infrared Spectroscopy (FTIR) spectra that the intensity of the phosphate and carbonate peaks diminished after acid etching, and the phosphate peaks appeared again and reached a high intensity plateau after 30 to 60 s treatments with NaOClaq. Research showed that sodium hypochlorite treatment exposes a labyrinth of lateral secondary tubules (Fig. 2) (3), which were not observed on etched dentin surfaces (Fig. 1), that could cause a rough topography of a surface may facilitate adhesives’ spreading and substrate’s wettability (20). Dentin permeability was also enhanced upon removal of organic materials (21). As other researchers (6,7), the NaOClaq solution application altered the ultra-morphology of etched dentin surface exposing a network of secondary lateral canals on superficial dentin, and widening the aperture of the dentin tubules (Fig. 2) on deep dentin (3), which should produce longer tags, related with a higher number of mixed failures ( Table 1).

PB NT is an acetone/based etch-and-rinse adhesive that contains an acidic phosphonated monomer (PENTA: dipentaerythritol pentaacrylate monophosphate), which also interacts with the calcium ions left on dentin surface even after collagen removal (22). Elastomeric dimethacrylate resins are also included in the formulation of PB NT and could compensate for polymerization contraction of the resin composite, thereby stabilizing bonding (10). Thus, after sodium hypochlorite treatment, an increase in SBS is expected. However, these facts might have been compensated by: (1) NaOCl, apart from being an effective deproteinizing agent, is also a potent biological oxidant (8), and in aqueous solution superoxide radicals -O2- are formed (23); (2) disruption by NaOCl of pyridoline cross-links that occur in the Type I dentin collagen (23) with the formation of chloramines and protein-derived radical intermediates (8); and (3) occurrence of residual glycosaminoglycan components of the organic matrix which are resistant to strong acids and NaOCl (8). The presence of these reactive residual free-radicals in NaOCl-treated dentin may compete with the propagating vinyl free-radicals generated during light-activation of the adhesive, resulting in premature chain termination and consequent incomplete polymerization (24). In addition, in the case of acidic methacrylate phosphates, as PENTA, an additional hydrolytic instability results from the hydrolysis of the methacrylate ester bond taking place in the presence of water (25).

As stated above, Prime and Bond NT is an acetone-based adhesive system. Its solvent is considered a good “water-chaser” and seems to have displaced water effectively from the dentin surface (10,26), resulting in an optimal infiltration into the treated substrate, even in the presence of perfusion through dentin (27). However, acetone-based adhesives are more technique sensitive to the degree of residual surface moisture, and this fact difficult to standardize the level of moisture left on the surface (6,26). In addition, its high volatility may also lead to reduce shelf life of acetone-containing adhesives, by rapid evaporation of the solvent (10). Manso et al. (28) concluded that the higher acetone content in this system resulted in lower values of bond strength.

Consistent with our PB NT observed contact angle data (29), the PB NT bonding effectiveness of resin was not influenced by NaOClaq treatment. Most likely, the nanofillers in PB NT composition reinforce the hypothe-sis that filler may reduce adhesive penetration into the etched (26) and NaOClaq treated dentin. Several authors (10) suggested that filler in the adhesive resin might decrease the wetting of the primed dentin surface because of the higher viscosity of filled resins (Fig. 2), thus reducing the shear bond strength in their studies. In this study, the filled adhesive formed thick layers (Fig. 2). The demineralized or collagen-depleted dentin may act as a sieve, with filler accumulating on the top and obstructing resin penetration into the dentin below (6,26). However, the presence of fillers may be important to produce a sufficiently thick resin film on the top of the hybrid layer (10). Even, if the nanofillers of the adhesive resin do not infiltrate inside the collagen fibril network, they may help to establish a uniform resin film that stabilizes the hybrid layer (30).

The results of the present study support the previous findings (6) in order that the qualitative and quantitative role of collagen fibers in optimizing adhesion must be questioned. The null hypothesis can only be confirmed in part. The application of a 5% NaOClaq solution for 2 minutes after dentin demineralization when PB NT was employed did not improve the bond strength to dentin. However, SEM findings show for NaOCl treated specimens that the hybrid layer was not apparent, and the neck of the cylindrical resin tags contacts directly with the adhesive. The presence of nanofiller in PB NT or oxidative changes on collagen-depleted dentin might have influenced the absence of variation on SBS measurements. Clinical studies should be performed prior to recommending the application of NaOCl on a routine basis with PB NT as adhesive system.
